# The Hospital Nursing Supplement

**Published:** 1895-10-19

**Authors:** 


### "The Hospital" Nursing Mirror.

Being the Extra Nursing Supplement of "The Hospital" Newspaper.

[Contributions for this Supplement should be addressed to the Editor, The Hospital, 428, Strand, London, W.C., and should have the word "Nursing" plainly written in left-hand top corner of the envelope.]

### News from the Nursing World.

### A BRAVE NURSE REWARDED.

Many of Miss Mary Ellen Barker's fellow nurses wll hear with pleasure of the gratifying recognition accorded to her courage by the "Royal Society for the Protection of Life from Fire." The medal is a particularly handsome one in silver, bearing on one side the name of the society, with the motto "Duty and Honour," and on the other a fine raised group of figures. Round the edge runs the name of the nurse and the date of the fire at Whiteway Chudleigh, when she by her promptitude and courage saved the life of the Dowager Countess of Morley. We believe that this is the first occasion on which this society has bestowed a medal on a nurse, and the honour was quite unanticipated by Miss Barker. Lady Morley presented her with a handsome case of instruments in token of her gratitude, and many kind acknowledgments of her bravery were volunteered by members of her ladyship's family. The fire, which accidentally occurred in the bed-room, was discovered by the nurse, who finding it gaining ground too rapidly to be subdued by the water she threw on the flames, lifted the helpless patient in her arms and carried her to an adjoining room. To the training gained during five years spent at the Nightingale School at St. Thomas's Hospital, Nurse Barker entirely attributes the possession of skill which enabled her to move, single-handed, a tall, heavy, and helpless lady; and to the same valuable experience she gives the credit of the admirable presence of mind which saved a human life. By promptly closing up the burning room, and summoning assistance, it is believed that much valuable property was also saved from destruction by a nurse of whom her friends may indeed feel proud.

### NURSES AT THE TEMPERANCE HOSPITAL.

The nurses at the London Temperance Hospital are particularly neat and trim, and their wards have an equally well-ordered appearance. In the latter, several inventions have been introduced by different sisters to facilitate the details of nursing. The iron rods used for bronchitis tents in the adult ward are, by an ingenious contrivance, invented by one of the Sisters, fitted rapidly to any bedstead by the turning of two screws, the simplicity and efficacy of the arrangement contrasting favourably with the obsolete form of the tents once universally used. A glass cover for the dressers' table to protect everything kept on it from dust has been introduced by another ward sister, and has a charming appearance. Each member of the nursing staff has a separate bedroom, and the common sitting-room contains a cottage piano and is most tastefully furnished. The nurses are encouraged to go out as much as possible by Miss Orme, who is certainly to be congratulated on the admirable condition of the hospital, which is fortunate in possessing so indefatigable a matron.

### OUR NEEDLEWORK COMPETITION.

We hope our readers are going to work very energetically during the next few weeks in making garments for distribution to those patients who spend Christmas Day in hospital wards. So many people complain of want of time that we propose this year to put into communication with each other Readers who have materials, but no leisure to make them up, and Readers with small means but plenty of leisure. Any communication on this subject should be addressed "Needlework," care of Editor of the Hospital, 428, Strand, London. The prizes offered are: One guinea for the best flannel dressing-gown, 10s. for best bed jacket (for man or woman), 10s. for best flannel shirt, 5s. for best flannel petticoat, 5s. for best over-petticoat, 5s. for best knitted socks, 2s. 6d. for second best pair, 2s. 6d. for best warm vest (for man or woman). Other kinds of garments can also be sent to us for distribution and should be marked "Not for competition."

### NURSES AT GREENWICH UNION.

The nurses on the female side of the Greenwich Workhouse presented a handsome brass inkstand to Dr. M. J. C. Keats on the occasion of his exchanging the position he has held for four years for one on the male side of the institution. The Sisters and nurses of the Union Infirmary (female side) gave, at the same time, a handsome toilet set of silver and tortoiseshell in token of their esteem and gratitude for Dr. Keats' constant help and encouragement to the nursing staff.

### THE WHITTUCK CONVALESCENT HOME.

A convalescent home has been opened at Broadstairs in connection with the Home and Hospital for Sick Children at Lower Sydenham. The new building, which is charmingly situated on the West Cliff, can accommodate twelve children, and it has been built, furnished and fitted at the expense of Mr. and Mrs. Whittuck Rabbits, who presented it as a free gift to the hospital, to which it must prove a valuable adjunct.

### A COTTAGE HOSPITAL FOR ACCRINGTON.

Much energy has been displayed in the neighbourhood of Accrington with regard to the proposed cottage hospital. Not only have the Women's Liberal Association organised an excellent bazaar, but working men and children of all classes have contributed such sums as they could. The exhibition of such general interest in the hospital, of which the foundation-stone is about to be laid, augurs well for its future support.

### A BREACH OF DISCIPLINE AT LINCOLN.

The Nursing Committee at Lincoln Workhouse reported at a recent meeting of the Guardians that a nurse in their employ "had left her duties without permission, to nurse her brother." The local press account contains no explanation of this extraordinary behaviour, and we await with interest the report of the next Board meeting, when it is to be hoped some light will be thrown on Nurse Clapham's action in deserting her responsible post. The provision of good nursing for paupers and the exercise of due consideration for nurses themselves are points which we unweariedly insist upon, and we still hope that the nurse may offer an explanation which will exonerate her from the blame which apparently attaches to her conduct, Had she been refused permission to go to her sick brother?

### NURSING AT BARRY DOCK.

With the work of the Barry, Cadoxton, and District Nursing Association many of our readers are familiar, and the urgent need for increased support should elicit a prompt response. It must be realised that many serious cases of illness have to be nursed at home, because the only general hospital in the district is many miles away. There is much local appreciation of the work of the nurses, and the bazaar fixed for December 9th, 10th, and 11th should add substantially to the funds of the association.

### GAINSBOROUGH NURSING ASSOCIATION.

The energetic members of the Britannia Band canvassed half the Gainsborough district the other day on behalf of the local Nursing Association. The collection resulted in £20 18s. being handed over to the charity, and Mr. James Marshall, J.P., kindly entertained the Band at tea afterwards, an attention which was thoroughly appreciated. The Gainsborough Nursing Association has evidently won the cordial interest of the neighbourhood.

### VICTORIA HOME, CHELTENHAM.

Miss Piggott, superintendent of the Victoria Home, Cheltenham, is resigning the post which she has filled so well to take up work at Sukkur, India, under the C.E.Z. Missionary Society. The departure of Miss Piggott from Cheltenham is deeply regretted by her friends, nurses, and a number of old patients to whom she has greatly endeared herself. Beginning with a small house and staff of four nurses, the home now contains about a dozen nurses (including the pupils) and a small maternity ward for in-patients. Miss Piggott is to be congratulated on the excellent work organised by her at Cheltenham, and her friends wish her equal success in a new country.

### NIGHT NURSING AT COVENTRY.

Public opinion at Coventry continues strongly in favour of the introduction of efficient night nurses at the workhouse infirmary, and the action of the Guardians who opposed their appointment has been again openly condemned. Among the citizens who have had the courage to advocate the rights of the sick poor are the members of the Hill-fields District Working Men's Gladstonian Club, and the local press reports that the following resolution was passed by them at a recent meeting: "That this meeting strongly protests against the action of those members of the Board of Guardians who voted against the proposal to appoint a night nurse at the workhouse infirmary, and we are of opinion that there is the necessity for such an appointment, not only for the sake of humanity, but to ensure that the aged and infirm should have skilled assistance rendered to them in the hour of need." This resolution was communicated to the Guardians at the next meeting of the Board, and was not discussed, although a member afterwards gave notice that he proposed to "have something to say about the nursing of the sick poor in the workhouse" in a fortnight's time.

### FORFAR DISTRICT NURSING ASSOCIATION.

The third annual report of this association shows that 120 patients have been nursed in their own homes during the past year, 2,660 visits being paid to them by the nurse. Many of the cases were serious, only twenty out of the whole number coming under the head of chronics. Miss Haddon, the assistant superintendent of the Central Home at Edinburgh, inspected the nursing at Forfar last spring, and reported favourably of the work and management. A nurse from Dundee was engaged to take the nurse's place during her holiday in August, and the committee appear anxious to consider in every way the comfort of the poor on whose behalf the Forfar District Nursing Association was called into existence.

### QUEEN'S NURSES AT ABERDEEN.

The bazaar opened by Lady Gordon Cathcart in Aberdeen has proved a most brilliant success. The proceeds of the two days' sale will not only cover the expense of furnishing the nurses' central home (Ingleborough House), but will also permit of a much-needed additional nurse being engaged by the District Nursing Association. Rapid progress has been already made by this association, which is affiliated with the Scotch branch of the Queen's Jubilee Institute, and the benefits conferred on the sick poor are fully appreciated at Aberdeen.

### AN AMERICAN DOOR PORTER.

A novelty has been introduced at Massachusetts in the form of a trained nurse as door porter at the General Hospital in that city. Her office is placed close to the front door, and she manages telephone, visitors, letters, and inquiries. The nurse has four male porters under her directions, and takes her own meals with the other nurses; she lives outside the hospital, is on duty from nine a.m. to five p.m., and is paid 50 dollars a month.

### SHORT ITEMS.

The Toxteth Guardians report favourably of the success attending the training of probationers in the Smithdown Road Workhouse Infirmary; certificates gained at the recent examination were presented to the successful candidates by the committee.—Mr. Alderman Domville will give six free lectures at Exeter on "Sick Nursing" to the students of the Technical and University Extension College.—Two concerts have been given at Mickleton in aid of the funds of the Nursing Association.—An under nurse at Ipswich Borough Asylum has been fined £2, and 12s. costs, for alleged ill-treatment of a patient.—A cot at the North-West London Hospital, Kentish Town Road, has been endowed by "The Children's Salon," and, by permission of Princess Christian, will be named "The Helena Cot."—Three nurses from Sunderland Union Infirmary successfully passed the examination of the London Obstetric Society, which they themselves considered was largely due to the excellent preliminary instruction they received from Dr. Prowde, the medical officer, and the lady superintendent, Miss Cowan.

### Elementary Physiology for Nurses.

By C. F. Marshall, late Surgical Registrar Hospital for Sick Children, Great Ormond Street.

### XI.—THE DIGESTIVE SYSTEM (*concluded*).

### The Gastric Juice.

The digestive action of the gastric juice is due to two factors—hydrochloric acid and pepsin. If a small piece of the fresh stomach of an animal is put in a tube with acidulated water and pieces of white of egg or meat and kept warm, the latter will become digested. The gastric juice converts proteids into soluble substances known as peptones, which can pass through the walls of the stomach into the blood. It has no action on fats or amyloids. In the stomach the food is thus reduced to the consistency of pea-soup, and is known as chyme; part of this is at once absorbed by the blood vessels and part is sent on into the duodenum.

The pancreatic juice is alkaline and is the most important of all the digestive juices. It acts on all the food-stuffs, converting proteids into peptones, starch into sugar, and also digests fat by a process known as emulsification, by which the fat is broken up into very minute particles.

The bile is alkaline, and is constantly being formed. It is not wholly digestive, but is largely excretory in nature as shown by the evil effects of its retention in the system, known as jaundice. Its main actions are to aid in the emulsification of fats, and to neutralise acidity. It also has an antiseptic action. Its use has, however, been probably overrated, for it has been shown that a man can get along very well when all the bile passes outside his body without entering the intestines, as sometimes happens in operations on the gall bladder for obstructive disease of the bile duct.

The intestinal juice is secreted by the glands in the intestine. Its action is similar to the pancreatic juice, but less active.

### The Absorption of Digested Fluid.

The mucous membrane of the small intestine is set with minute club-shaped processes or villi, like the pile of velvet. Inside each of the villi is a lacteal or lymphatic vessel. The lacteals absorb fat from the food, and the milky fluid in the lacteals is called chyle. The chyle is carried to the lymphatic vessels, and ultimately to the thoracic duct; and so into the venous system. Surrounding the lacteal vessel is a network of capillaries of the arterial system, and into these peptones and sugars are absorbed from the food. These are carried to the liver by the portal vein. In the large intestines the watery parts of the food become rapidly absorbed, and the contents assume the appearance of fæces.

*The Action of the Liver.*—It is important to note that all the blood from the alimentary canal goes to and through the liver before reaching the heart. What does it do in the liver? In this organ large quantities of a substance called glycogen are formed; this substance is readily converted into sugar, and is probably of use to the system.

*Assimilation.*—This is the process by which living organisms are capable of incorporating the substances obtained from their food into their own tissue, and making them an integral part of their own bodies. This process is probably effected in the cells of the tissues themselves. The substance required for assimilation may be obtained from animals or plants. The proportions of the constituents in vegetable foods more nearly approach the required one than do animal foods. Speaking generally, vegetable foods are relatively deficient in nitrogen, and animal foods in carbon. A judicious combination of the two affords the requisite quantities, with the least excess of any one; *i.e.*, with the least waste of energy on the part of the digestive organs. The diet best suited to the body should contain one part of nitrogenous food to $3\frac{1}{2}$—$4\frac{1}{2}$ of non-nitrogenous. It would be a great mistake to live on beef alone or on potatoes alone. The relative amounts of nitrogenous and non-nitrogenous parts in various foods is shown in the following table:—

| | Nitrogenous. | Non-nitrogenous. |
|---|---|---|
| Veal ... ... ... | 10 ... | ... 1 |
| Beef ... ... ... | 10 ... | ... 17 |
| Mutton ... ... | 10 ... | ... 27 |
| Cow's milk ... ... | 10 ... | ... 30 |
| Human milk ... ... | 10 ... | ... 37 |
| Wheaten flower ... | 10 ... | ... 46 |
| Oatmeal ... ... | 10 ... | ... 50 |
| Potatoes ... ... | 10 ... | ... 100 |

Let us conclude this lecture with a consideration of what happens during the digestion of a meal of beef-steak, potatoes, bread, and water. (1) The beef is first masticated in the mouth and then mixed with the saliva, but no digestion takes place till it reaches the stomach. Here it is attacked by the gastric juice and broken up and dissolved. By the movements of the stomach all parts of the food are rolled about and come in contact with its walls. The proteids of the meat are converted into peptones and taken up by the capillaries of the stomach. From the stomach the partly digested matter is squeezed into the intestine, and attacked by the pancreatic juice, and more peptones formed and absorbed by the intestines. The fat of the beef is not acted on till it reaches the duodendum. There it is broken up by the pancreatic juice and bile into tiny drops, which are absorbed by the lacteal vessels in the villi of the intestine. The salts in the meat, being soluble, are absorbed quickly. (2) Potatoes: These contain a great deal of starch, a little proteid, some salts, and a trace of fat. The starch is at once converted into sugar by the saliva in the mouth, and again by the pancreatic juice in the intestine. The digestion of the proteid part of the potatoes is similar to that of the meat. (3) Bread: This contains about 50 per cent. of starchy matter, and 8 per cent. of proteids, also some fat and salts. The digestion of these takes place in the same way as the above. The food absorbed by the lacteals is forced directly into the veins; that taken up by the blood-vessels is first carried to the liver, where it undergoes important changes, thence it is taken to the vena cava, and so to the right auricle of the heart.

### Notes and Queries.

### Queries.

(7) *Tynemouth Victoria Jubilee Infirmary.*—I shall be glad if you can tell me through The Hospital if it is usual in small provincial infirmaries for the committee to appoint an honorary superintendent. If so, what will be considered his duties, where there is no resident surgeon, a paid secretary, and the matron held responsible for the observance of every order and regulation connected with the wards, the nurses, and the servants?—*H. E. A.*

(8) *Drugs.*—A cheap and concise book on drugs wanted by a nurse; please advise.—*T. D. S.*

(9) *Midwifery.*—Please recommend a book for a midwife.

(10) *Dispensary.*—How long is required to qualify as a dispenser?—*Minon.*

(11) *Irish.*—What dress and badge does an Irish nurse wear? It is wanted for a fancy dress bazaar.—*Nurse S.*

(12) *Male Nurse.*—Where can I get trained as a male nurse?—*E. S.*

### Answers.

(7) *Tynemouth Victoria Jubilee Infirmary* (*H.E.A.*).—The duties of an honorary superintendent of a small hospital will be found in "Cottage Hospitals," by Henry C. Burdett. The holder of this position is usually a medical man, and in several cases the superintendent has also acted as honorary secretary to the institution. In this capacity he has the general supervision of all the departments of the institution, and exercises control over the various officials employed therein.

(8) *Drugs* (*T. D. S.*)—If you are studying for a dispenser read article in The Hospital of September 24th, 1892, p. clxxxiii. Various books are named there.

(9) *Midwifery.*—"Handbook for Mothers," by Jane H. Walker, M.D.; "Practical Handbook of Midwifery," by Dr. F. W. N. Haultain.

(10) *Dispensary* (*Minon*).—See answer to No. 8 this week.

(11) *Irish* (*Nurse S.*)—There is no Irish nursing uniform, any more than there is a universal English one. Each hospital has its own.

(12) *Male Nurse* (*E. S.*) —No English general hospital trains male nurses.

### Neuralgia and its Treatment.

By Rowland Humphreys, M.R.C.S., L.R.C.P., &c.

II.

Another severe form of neuralgia is that known as angina pectoris, pain affecting the heart, and often associated with disease of the vessels which supply the heart's muscle; or it may be due to the increased strain suddenly thrown on the heart if the smaller arteries suddenly contract. The treatment for it lies in relieving the strain thrown on the heart by the administration of amyl nitrite as an inhalation or of nitro-glycerine internally. Both these drugs cause rapid and general dilatation of the blood vessels of the body, and so relieve the tension in them.

Another form of neuralgia is that of the ovary, to which many persons are subject at each recurrence of the periods. This is too often a very difficult matter to deal with, but the external application of mustard, of poultices, of soothing applications will often relieve, and with the progress of the cause the pain generally departs. The drugs already mentioned do not appear to relieve as much as might have been expected, and there is a tendency to dose the patient with gin and similar stimulants in order to relieve the immediate trouble, too often leaving behind them a taste for alcoholic drinks. They will also do little to relieve the pain, and a dose of sweet spirits of nitre (a teapoonful), or a very hot hip bath for twenty minutes on going to bed, will do far more. Neuralgia of this organ occurring at other times may often be relieved by the use of the liquid extract of the black willow, and as this occurs more especially at the "change of life" the nerve symptoms usually superadded, but traceable to the ovarian pain, may often require medical treatment, combined with extra feeding.

Extra feeding, such as is systematically employed for the treatment of neurasthenic cases, is of the greatest service in these, as in all cases of neuralgia of long standing. These patients as a rule eat badly, and this is especially the case in the abdominal cases, where the pain is increased by the ingestion of food. The best rule in their treatment is that in a chronic case the patient must take food in sufficient quantity to satisfy a male adult doing hard work. This stage will not be reached in a day, and the teaching of the patient to regard her bodily necessities rather than her sensations is a very necessary part of it. Neuralgia is to be treated by food and not by drugs. Immediate relief must be given if the patient is to have any faith in the treatment; it is quite as useless to talk to a drunken man as to instruct a person suffering from a severe neuralgia in the art of avoiding a recurrence of the trouble till she has had her sufferings relieved. The introduction and popularising of antipyrin, &c., is not altogether a lightening of the task to either nurse or patient.

The treatment of neuralgia resolves itself into relief of the pain for the moment, the removal of the exciting cause, and the strengthening of the body generally. Pain may be relieved by the doctor prescribing one of the numerous r[illegible]edies now to be had for this purpose, such as antipyrin, antifebrin, phenacetin, or, if the case is a very bad or a long continued one, it is best to begin with a small dose of morphia, or an injection of cocaine near the seat of the pain, and the action of these drugs may be continued by the use of one of the other drugs just mentioned. In some cases, where the patient is rheumatic, the administration of salicylate of soda will relieve more than anything else. Sometimes external applications will relieve or will assist the action of the drugs given internally. One of the best of these is a mixture of equal quantities of menthol and butyl-chloral-hydrate, which dissolve one another, with some spirits of wine added. Another application is very hot water, constantly applied for some length of time. Another remedy in intercostal and in spinal neuralgias is counter-irritation (in the form of a part of a mustard leaf or a blister) to the skin near the spine or over one of the branches of the nerve. As the patient gets better, the nurse is often requested to put the leaf on the actual seat of the pain, but this does not appear to yield much relief in the worst stages; indeed, it often causes pain to be worse.

As has been said, neuralgia may make itself felt along the course of any nerve, and thus it is often felt in the arms, usually the lower arm. This form of neuralgia is often relieved by the galvanic or Faradic current, and this may succeed when all other remedies fail.

In all forms of neuralgia cod-liver oil is of the greatest service, and, if unable to take it, cream, milk in good quantity, and similar articles of diet may be substituted for it. The patient should commence the day by taking a glass of warm milk before getting up, and at eleven a.m. and four p.m. should have either an egg, two or three meat sandwiches, or some nutritive beef-tea. At the ordinary meal hours she should take a light meal consisting of several things, rather than to fatigue the appetite with a large meal off one dish. She should also take some soup on going to bed, and have something alongside the bed to take in the night if at all wakeful, or should even be waked up for the purpose.

Stimulants in small quantity may be taken at meal time, but only as an adjunct to food. A glass of port wine will often relieve pain for a while, as it enables the person to go on longer, but if taken by itself it means an increased loss of power, made less noticeable for the moment by the stimulating influence of the half-ounce or more of alcohol in the wine.

The action of the bowels must be carefully regulated. In many cases of neuralgia the immediate cause of the pain appears to be a contraction of the arteries supplying the affected nerve, the contraction being caused by the presence of irritating substances in the blood, which have been absorbed from the intestines and which have escaped destruction in the liver and lungs. The administration by the mouth of intestinal antiseptics will also assist by preventing the formation of these poisonous substances.

Any dyspeptic condition should be carefully treated, as it means a failure to absorb the nourishment placed in the digestive organs, and so prevents the proper supply of food to the tissues.

A glass of very hot water, slowly sipped, two or three times a day at meals, or between them, will greatly assist most forms of indigestion.

### Novelties for Nurses.

### AUSTRALLAMA WOOLLEN MATERIALS.

We have received a very comprehensive catalogue from Messrs. Greensmith and Downes, of George Street, Edinburgh, in which full particulars, illustrated, are given of their specialities for underclothing. We notice in particular a very nice bodice petticoat, which is convenient to wear and very warm. The sample material attached to the catalogue is beautifully soft, well woven, and elastic.

### NURSES' WANTS AND SICK-ROOM APPLIANCES.

The above is the title which Messrs. Hockin and Wilson, of Tottenham Court Road, have adopted for their excellent little catalogue of necessaries for the sick-room. The catalogue is fully illustrated, and will prove most useful when a purchase has to be made. From thermometers to cooking utensils nothing appears to have been forgotten which can add to the convenience of nursing.

### Where to Go.

Trained Nurses' Club, 12, Buckingham Street, Strand.—*Nursing Notes*' Exhibition of Nursing Appliances, which will open at this address on Friday, 18th inst. (admission 6d.), must prove of particular interest to nurses.

Exhibition of the Society of Portrait Painters, New Gallery.—Taken as a whole, the portraiture at the New Gallery is a good deal above the average, although there are few really remarkable pictures. The standard is by no means a low one, and there is but little bad work. In the present exhibition it is certainly the good work, and not the bad, which is noticeable. Would that one could say this oftener of our annual picture shows. Among these we recognise many old friends, and feel small regret at another meeting with the Hon. John Collier's "Miss Julia Neilson," and others; but it is over the newer works one lingers with greater interest. Mr. Collier is represented by five other canvasses. His "Professor Huxley," painted in 1883, has toned down and improved since we saw it last. Carolus Duran, it is to be regretted, shows only one picture—"Fritz Thaulow" (No. 59)—in the north room. This same room has on its western wall a perfect gallery of child portraiture; with "Fair Children" but freshly in our minds this is especially noticeable. Very sweet they are, some of these picture. Further along is Sily Dellesa's portrait of "The Painter," one of the canvasses to be singled out in this exhibition. But it is a question of taste whether this is singled out for unqualified admiration—as a work of art it deserves praise, as it shows an undoubted technical power and a certain dramatic force which raises it above its surroundings. And speaking of dramatic force, where in this gallery is it better displayed than in Sarah Bernhardt's portrait by Antoine Gandara? The actress is represented standing with her back to the spectator, as one has seen her many a time in real life, the head turned a little over the shoulder; the position is caught cleverly and characteristically. One feels in the actual presence of the divine Sara. The background is simple, the painting-in of the figure simple, and nothing of affectation in technique distracts one's eye. Prince Troubetzkoy has two studies in the west room; we call them studies, but more definitely speaking they are portraits. This artist, who paints after the method of the Impressionist School, one instinctively feels is trying to express something in his work beyond an actual representation of the physique of his sitters. The inevitable portraits *a la* Sir Joshua Reynolds are to be found in Mr. Ellis Roberts' life-size paintings of "The Countess of Yarborough" and "The Duchess of Portland." These occupy a conspicuous portion in the north room. The two women are standing in bowery surroundings, as many other fair ladies stand in the canvas of the great Sir Joshua Reynolds. Opposite to these there is a smaller painting by Gustave Courtois (No. 106) called A Portrait." Here we have an example of individualism in brush work. This has nothing of the flesh and blood of human life about it, but is suggestive of statuary on canvas, and is unique if nothing else. We must not omit to mention another remarkable painting, also of a unique order, by L. W. Hawkins. "Madame Severine" (No. 146) is an example of the striking if not the pleasing in art. But this compels one to look at it a second and a third time. It is a portrait of a woman of genius, and the curious mesmerism which the woman exercised in life the painter has transmitted in an unaccountable manner to his canvas, it is, as perhaps it should be, a remarkable treatment of a remarkable woman, and Mr. Hawkins has applied an intellectual method in his work. One looks in vain round these walls for any examples of Serjeant's work, and insensibly their want is felt, and where are the canvases of the prolific and versatile Professor Herkomer? If one does not mourn their loss, one notices their absence.

### Our American Letter.

Contributed.

A gift of a very elegant bicycle was received by Miss Margaret Orr from the nurses at the Paterson General Hospital on her resignation of the post of superintendent of the Training School. Miss Orr has been deservedly popular with the graduates, to whose well-being she has devoted ten years.

Miss Gordon McIntosh, a graduate of the Scarritt Training School, is on her way to take up mission work in India. It is to be regretted that she will have to devote herself to acquiring the language of Hindostan on her arrival before she can commence training native nurses, which is one of her chief objects in going to India. The plan adopted by French and other missionary societies of instructing their pupils in the language of the country to which they are going, might advantageously be copied in the U.S.A.

At Worcester, Mass., yellow and white are the distinguishing colours of the graduates, eleven of whom recently obtained diplomas on the completion of two years' training. At the General Hospital, Winnipeg, a course of three years' training has been recently instituted.

At the Metropolitan Hospital in New York, which enjoys a high reputation as a training school, certificates are given at the end of two years. The death this summer in the Nurses' Home of Miss May Tyndall caused great regret and sorrow to her friends and fellow-workers. She was an English nurse, and had held for some years the position of head nurse at the Metropolitan Hospital, where she did valuable work which was thoroughly appreciated by all who knew her.

An Alumnæ Association has been recently formed by the nurses of the Maryland General Hospital, and has made a satisfactory beginning.

The Thomas Wilson Sanatorium is the scene of a wonderful and beautiful work, for it represents, some twelve miles from Baltimore, an ideal children's summer hospital. It is for little ones under five years of age, and they are received either with or without their mothers. If the mother has other children whom she cannot leave, they are permitted to accompany her and remain until the patient is discharged by the physician. Everything is done to restore health to the children, and they are fed, clothed, and transferred to and from the hospital free of expense.

A new arrangement has been introduced at the Massachusetts General Hospital with considerable success. This is the appointment of a trained nurse for "front-door service"—in other words, as head door-porter. Nurse Annie Durling has four male assistants, but she is personally responsible between the hours of nine and five for the administration of this department. She receives all messages and visitors, answers questions relating to patients, and sees to the distribution of letters, &c. This, probably, is the first time a woman has received an appointment of the kind.

### Minor Appointments.

Mirfield Memorial Hospital.—Miss Eline Traiforos has been appointed Nurse-Matron of this hospital. She was trained at the Victoria Hospital, Burnley, and was afterwards Sister-in-Charge at the Royal Infirmary, Aberdeen. We congratulate Miss Traiforos on her promotion, and wish her every success.

### Everybody's Opinion.

[Correspondence on all subjects is invited, but we cannot in any way be responsible for the opinions expressed by our correspondents. No communications can be entertained if the name and address of the correspondent is not given, or unless one side of the paper only be written on.]

### HOURS AND WORK OF ASYLUM ATTENDANTS.

"An Asylum Matron" writes: In The Hospital of October 5th there appeared a letter from "An Asylum Nurse" containing some strange and misleading statements. I have been for over twelve years matron of a large asylum, and I cannot think where the "Asylum Nurse" can have gained her experience when she speaks of being molested by patients at her meals, and kept up till ten, eleven, or twelve o'clock. In all well-conducted asylums the nurses have their meals quite apart from the patients, and the rule is that lights must be out at half-past ten. Patients go to bed from eight to ten o'clock. I have also to contradict her statement that asylum nurses are kept on their feet all day long. It may be so in the evidently badly-managed asylum where she has gained her experience, but in ordinary large asylums the nurses are sitting beside their charges almost the whole evening, and for many hours during the day. I have known many asylum nurses who have been sent, or have voluntarily gone, for hospital training, and they find invariably that the work is much harder in a hospital than in an asylum—that it is in the hospital, not the asylum, in which they have to be on their feet all day; and asylum nurses have the advantage of being out of doors for two to three hours daily walking with their charges. As a set-off to this, the mental strain is greater in asylum than in hospital work, as the patients are often extremely depressed, irritating, or require unceasing watchfulness lest they do harm to themselves or others. A perfect nurse ought to have both hospital and asylum training, and ought to feel so much enthusiasm in her profession that she cares not how much trouble her patients give her if she can have the satisfaction of seeing them gradually regain health. The "Asylum Nurse" who wrote in The Hospital of October 5th has evidently mistaken her vocation, as she even thinks it a grievance to be expected to gain a certificate!

"A Doll" writes: In reply to letters in your issues of the 5th and 12th, I beg to state that, as an attendant of 15 years' standing, I can truthfully say that there is real need for some improvement in our social position, both as regards hours of duty, pay, and pension. I certainly disagree with Dr. Urquhart's opinion that the nurse's letter of the 5th damages our cause. It is by making known our grievances that we hope to get them redressed. That writer has given expression to truths which (if it were not for the fear of a few doctors) a good many other nurses and attendants would corroborate and augment to such an extent that the medical profession and the nursing world would not want to study the ratepayers in the same way as is done at present—short pay for attendants, to keep and clothe a family on; and then, having saved so much, the ratepayers are asked for a rise of salary for the superintendent to the tune of £200 per annum. The attendant gets three-halfpence per hour. The Doctor forgets there is a duty owing to the attendant. In connection with the murder at Cane Hill Asylum, it was stated that three attendants are sufficient for 100 patients in the airing courts! With reference to the psychological certificate, in every branch where badges are worn they carry remuneration with them. The asylum doll, dressed with badges and medals, receives sympathy (which is cheap) from those who should also champion her cause. I trust, sir, as a wearer of the above, you will grant me space in your columns.

### ASYLUM LIFE.

"Two Asylum Nurses" write: Since one of our sisters claimed a small space to tell her tale of woe in The Hospital, kindly allow us the same privilege to eradicate the poor opinion she gives the public respecting "Asylum life." The duties to be performed are many; but with good management all menial work should be finished by noon after which lighter duties follow. The calling of an asylum nurse is honourable rather than despicable, since from experience we know the care and patience the insane require both in good and ill-health. Certainly we are on duty a good many hours. As all struggles are avoided as much as possible we cannot imagine a nurse allowed to struggle with a patient from six a.m. till ten p.m. It is usual for nurses to relieve each other with refractory cases. Should we receive a blow, both doctors and matron sympathise with us, which is all that we can expect, for patients are not in asylums to be punished, but to be taken care of. For anyone entertaining such thoughts as were expressed in last week's letter, to remain in asylum life is doing an injustice to herself; since the necessary self-sacrifice is so grudgingly given. No medical superintendent can compel a nurse to remain longer than she chooses. The amusements provided for the patients may be considered a pleasure rather than duty. We are more fortunate than girls in business where they have to stand the greater part of the day. Then why grumble?

### NURSES' DIET IN INFIRMARIES.

"A Working Nurse" writes: Will you do what lies in your power to remedy the bad feeding for the nurses at the East Dulwich Infirmary. Is it the fault of the contractors or the hospital authorities? Surely women who labour as they do are at least entitled to the plain and wholesome food which every good master will give to his hard-worked cattle. Probationers are constantly leaving, and from my knowledge of them I feel sure it is not always the hard work.

### REAL AND SHAM NURSES.

"A Provincial Trained Nurse" writes: I would like very much to protest against the use, or rather mis-use, of nurses' uniform by other than *bona fide* nurses; the practice is fast bringing it into disrepute and losing for it its time-honoured respect. Any person may (and I am given to understand that numbers do) adopt a nurse's uniform, although probably she has never had a day's training in a hospital ward. But this is only a minor detail. So long as any woman chooses to masquerade in borrowed plumes, she may do so with impunity, and under the existing state of affairs there is no power to prevent her. Servant girls, housemaids, nursemaids, women of questionable character, anybody and everybody, if they think it ministers to their vanity or attracts attention, wear it without fear of unpleasant consequences. It is different if a civilian masquerades in a soldier's uniform. If detected, he is fined or imprisoned by law; why should it not be made a punishable offence to wear a nurse's uniform? I contend that although we do not actually fight the enemy with sword and gun, we do with treatment and method, under the command of our medical men; nor do we hesitate to risk our health and lives by daily contact with those who are suffering from malignant or contagious diseases. So long as nurses choose to remain as they are, and make no combined effort to prevent the abuse of their uniform, so long may they expect treatment such as your correspondent described to us recently. Our most gracious Queen, and H.R.H. the Princess of Wales, as well as many others of the Royal Family and aristocracy of our land, have repeatedly shown the kindly interest they take in nurses. I am sure if a representation was made in the proper quarter, sympathy and aid would speedily be enlisted, and it would soon become no longer possible for anybody and everybody to wear a nurse's uniform. I am sure that if once a motion was set on foot, all nurses would join in it heartily.

